# Enrichment of MTHFR 677 T in a Chinese long-lived cohort and its association with lipid modulation

**DOI:** 10.1186/1476-511X-13-104

**Published:** 2014-06-26

**Authors:** Ning-Yuan Chen, Cheng-Wu Liu, Li-Li Du, Li-Ping Xiao, Lin Ge, Yi-Yuan Wang, Zhen Wei, Hua-Yu Wu, Chen-Yuan Luo, Liang Liang, Jun-Hua Peng, Xiao-Qiu Luo, Rui-Xing Yin, Cuc Phuong Nguyen, Shang-Ling Pan

**Affiliations:** 1 Department of Pathophysiology, School of Preclinical Medicine, Guangxi Medical University, Nanning 530021, Guangxi, People’s Republic of China; 2 Department of Cell Biology & Genetics, School of Preclinical Medicine, Guangxi Medical University, Nanning 530021, Guangxi, People’s Republic of China; 3 Department of Cardiology, Institute of Cardiovascular Diseases, The First Affiliated Hospital, Guangxi Medical University, 22 Shuangyong Road, Nanning 530021, Guangxi, People’s Republic of China

## Abstract

**Background:**

Variants in the Methylenetetrahydrofolate reductase (MTHFR) gene may result in a lowered catalytic activity and associate with subsequent elevated serum homocysteine (Hcy) concentration, abnormal DNA synthesis and methylation, cardiovascular risk, and unhealthy aging. Several investigations on the relationship of MTHFR C677T polymorphism with serum lipid profile and longevity have been conducted in some populations, but the findings remain mixed. Herein, we sought to look at the association between MTHFR C677T and lipid profile in a longevous cohort in Bama, a well-known home of longevity in China.

**Methods:**

Genotyping of MTHFR C677T was undertaken in 516 long-lived inhabitants (aged 90 and older, long-lived group, LG) and 493 healthy controls (aged 60–75, non-long-lived group, non-LG) recruited from Bama area. Correlation between MTHFR genotypes and lipids was then evaluated.

**Results:**

T allele and TT genotype were significantly more prevalent in LG (P = 0.001 and 0.002, respectively), especially in females, than in non-LG. No difference in the tested lipid measures among MTHFR C677T genotypes was observed in LG, non-LG and total population (P > 0.05 for all). However, female but not male T carriers exhibited higher TC and LDL-C levels than did T noncarriers in the total population and in LG after stratification by sex (P < 0.05 for each). These differences did not however remain through further subdivision by hyperlipidemia and normolipidemia.

**Conclusion:**

The higher prevalence of MTHFR 677 T genotypes and its modest unfavorable impact on lipids in Bama long-lived individuals may imply an existence of other protective genotypes which require further determination.

## Introduction

Methylenetetrahydrofolate reductase (MTHFR) catalyses the conversion of 5,10-methylenetetrahydrofolate to 5-methyltetrahydrofolate, the methyl donor which re-methylates homocysteine (Hcy) to methionine [1]. Methionine is the immediate precursor of S-adenosylmethionine (SAM), the universal methyl donor of numerous biological methylation reactions that are essential to the synthesis of DNA, proteins, phospholipids and various neurotransmitters [2]. Recently, the relation between Hcy and lipid metabolism has been underpinned, and the proposed biochemical link between the two is the SAM-dependent formation of phosphatidylcholine from phosphatidylethanolamine [3]. Phospholipids are associated with very low-density lipoprotein assembly, thus affecting the high-density lipoprotein (HDL) pool [4]. Many studies have demonstrated that insufficient MTHFR activity confers higher serum Hcy level which has been increasingly linked to the pathogenesis of several age-related disorders, such as inherited thrombophilias [5], ischemic heart disease, stroke [6], Alzheimer’s disease (AD) [7], cognition impairment [8], and diabetes [9]. The underlying mechanism of age-related phenotypes under higher Hcy condition may point to its detrimental damage on vascular endothelial cells due to uncontrolled lipid peroxidation, enhanced oxidative stress and inflammation [10,11]. Elevated serum Hcy concentration (hyperhomocysteinemia) can in the main be ascribed to vitamin deficiency (e.g. folate, B12 and B6), renal dysfunction, aging or a common variance to the MTHFR gene, in which cytosine is replaced by thymidine (C > T) at base position 677. This single nucleotide polymorphism (rs1801133) causes an alanine-to-valine substitution in the MTHFR protein at polypeptide position 222 that renders the enzyme more thermolabile and less active as compared to the wild-type enzyme [12].

Collectively, a complex relationship is potentially existed among Hcy, lipid levels, MTHFR C677T polymorphism, aging, and ultimately, lifespan. The exploration of the relation between the MTHFR C677T and lipid profiles in long-lived individuals may thus help to provide insight into the biology of human aging and aging-related diseases. However, available literatures from this subfield are limited and the results are mixed. Bama long-lived individuals, a unique cohort reside along the midstream of Hongshuihe River in Guangxi Province, P.R. China, has emerged as an optimal cohort for human aging/longevity study in view of its relatively uniform genetic background over the past decades [13]. We designed the present study to test the hypothesis that the MTHFR C677T polymorphism is associated with different serum lipid profiles and may partially account for the longevity in Bama nonagenarians/centenarians of Zhuang ethnic origin.

## Materials and methods

### Study subjects

We screened common age-related disorders including coronary heart disease (CHD), stroke, hypertension, diabetes, cancer, gout, asthma, chronic bronchitis and chronic sinusitis for 574 nonagenarians/centenarians who have been physically living in Bama area (Bama, Fengshan, Donglan, and Du’an County) along the midstream of Hongshuihe River Basin, Guangxi Zhuang Autonomous Region, the People’s Republic of China by two experienced physicians and excluded 58 individuals who were evidently unhealthy (stroke, n = 16; Parkinson disease, n = 2; hypertension, taking medication, n = 22; emphysema and chronic bronchitis, n = 9; long-term bedridden sufferers of unknown causes, n = 11). Finally, 516 apparently healthy nonagenarians/centenarians (127 males and 389 females, age 93.23 ± 2.95 and range 90–104 years) were included in this study (referred to hereafter as the long-lived group or LG). At the same time, 530 elderly aged 60–75 years without a familial history of exceptional longevity (no past or current nonagenarian/centenarian in the first, second and third degree relatives) from the same geographic region were also screened for the same spectrum of diseases, 493 of which (142 males and 351 females, age 67.19 ± 4.70 and range 60–75 years) were finally recruited as controls (non-long-lived group, non-LG) and 37 were excluded due to stroke (n = 2), hypertension (taking medication, n = 14), diabetes (taking medication, n = 3), chronic bronchitis (n = 10) and heavy alcoholics (n = 8). All subjects under investigation were unrelated and belong to Zhuang ethnic group, the China’s largest minority mainly residing in Guangxi. Approval for the current study was obtained from the Ethics Committee of Guangxi Medical University. All participants gave their written informed consent after an extensive description of the study aims and design.

### Epidemiological survey

Socio-demographic information was obtained using a standardized questionnaire. Anthropometric measures including height, weight and waist were measured in all groups. Body mass index (BMI) was calculated as weight (kg)/height^2^ (m). Sitting blood pressure measures (average of three readings) were collected using a standard mercury sphygmomanometer with the subject resting for at least 5 minutes before measurement. Systolic blood pressure was determined by the first Korotkoff sound; and diastolic, by the fifth Korotkoff sound. Hypertension was defined as systolic blood pressure > 140 mmHg and/or diastolic blood pressure > 90 mmHg. Normal weight, overweight, and obesity were defined as a BMI < 24, 24 to 28, and > 28 kg/m^2^, respectively [14].

### Biochemical measurements

A blood sample of 8 mL was collected by venipuncture from each subject after an overnight fast of > 8 h, 4 mL of which was for serum separation and subsequent lipid determination while the remaining was transferred to an anticoagulant tube (4.80 g/L citric acid, 14.70 g/L glucose, and 13.20 g/L trisodium citrate) for DNA isolation. Total cholesterol (TC), triglycerides (TG), LDL-C and high density lipoprotein cholesterol (HDL-C) concentrations were measured by standard enzymatic methods using commercially available kits (Daiichi Pure Chemicals Co, Ltd., Tokyo, Japan) on a biochemical analyzer (Type 7170A; Hitachi Ltd, Tokyo, Japan) at our Clinical Science Experimental Center. The normal ranges of serum TC, TG, HDL-C, and LDL-C levels in the Center were 3.10-5.17, 0.56-1.70, 0.91-1.81, and 1.70-3.20 mmol/L, respectively. The individuals with TC > 5.17 mmol/L and/or TG > 1.70 mmol/L were defined as dyslipidemia [15].

### Genotyping

Genomic DNA was isolated from nucleated blood cells using standard methods [16]. The nt 677 C > T variance was determined by use of the polymerase chain reaction (PCR) and HinfI restriction enzyme digestion as described by Frosst et al. [17]. Briefly, a 198-bp fragment in the exon 4 of the MTHFR gene was amplified by using primers 5'-TGA AGG AGA AGG TGT CTG CGG GA-3' (forward) and 5'-AGG ACG GTG CGG TGA GAG TG-3' (reverse) (Sangon Biotech, China). PCR was performed in a volume of 20 μL containing 200 ng of genomic DNA, 10 μL of 2× Taq MasterMix (Beijing CoWin Bioscience, China), 6.25 μM (1.0 μL) of each primer, 5 μL ddH2O and 1 U of DNA polymerase (Takara Biotechnology, DaLian, China). The mixture was initially denatured at 94°C for 2 min, followed by 33 cycles of 95°C 30 sec, 61°C 30 sec, and 72°C 30 sec, with a final 7 min extension at 72°C. The amplified PCR products (13 μL) were digested with *Hinf*I (5 U) restriction endonuclease (New England Biolabs, Beijing, China) at 37°C for 4 h, and the restriction digestion products were separated on a 3% agarose gel and visualized by ethidium bromide staining. The mutant allele (677 T) gives *Hinf*I restriction fragments of 175 bp and 23 bp, whereas the normal allele (C677), gives a single fragment of 198 bp. To assess genotyping reliability, six randomly selected DNA samples (two for each genotype) were directly sequenced and the sequencing results were all consistent with that of genotyping. Laboratory technicians who performed genotyping were blinded to clinical and biochemical data.

### Statistical analysis

Data were analyzed with the statistical package SPSS 13.0 (SPSS Inc, Chicago, IL). Levels of the quantitative variables are presented as mean ± SD. Allelic and genotypic frequencies were calculated directly. Comparison of values of general characteristics between study groups and test for Hardy-Weinberg equilibrium were performed with the Pearson chi-square test or analysis of covariance (ANCOVA). The statistical evaluation for the categorical variables was based on the calculation of the Student *t*-test. The association between the MTHFR C677T polymorphism and lipid variables was tested by ANCOVA. Multiple logistic analyses with stepwise modelling were used to evaluate the association of serum lipid levels with genotypes (CC = 1, CT = 2, TT = 3) and several environment factors. In all hypothesis tests, two-tailed values of P < 0.05 were considered statistically significant.

## Results

### General characteristics and serum lipid levels

The basic demographic, clinical and biochemical data of long-lived individuals and controls are presented in Table 1. After adjusting for covariates, BMI was significantly lower while the hypertension rate was significantly higher in LG as compared to non-LG group (*P* = 0.023 and *P* < 0.001 respectively). No statistical difference was observed on other measures between the two groups (P > 0.05 for all).

**Table 1:** Subject characteristics

	**LG (n = 516)**	**non-LG (n = 493)**	**F (t or χ**^**2**^**)**	***P***
Age (year)	93.23 ± 2.95	67.19 ± 4.70	104.823	0.000
Male/female	127/389	142/351	2.265	0.132
BMI (kg/m^2^)	20.40 ± 3.62	21.40 ± 3.11	5.161	0.023
SBP (mmHg)	166.72 ± 27.65	135.87 ± 23.25	1.927	0.165
DBP (mmHg)	89.36 ± 13.58	83.15 ± 11.10	0.931	0.335
TC (mmol/L)	5.15 ± 1.03	4.97 ± 0.96	0.522	0.470
TG (mmol/L)	0.98 (0.48)	0.91 (0.59)	1.979	0.160
HDL-C (mmol/L)	1.60 ± 0.38	1.71 ± 0.41	0.841	0.359
LDL-C (mmol/L)	3.05 ± 0.87	2.73 ± 0.88	0.018	0.892
Dyslipidemia n (%)	261 (50.58)	227 (46.04)	2.078	0.149
Hypertension n (%)	456 (88.37)	231 (46.86)	200.000	0.000

Note: LG, long-lived group; non-LG, non-long-lived group; BMI: body mass index; SBP, systolic blood pressure; DBP, diastolic blood pressure; TC, total cholesterol; TG, triglyceride; HDL-C, high-density lipoprotein cholesterol; LDL-C, low-density lipoprotein cholesterol. TG values were presented as median (interquartile range) while other measures were presented as mean ± SD. The difference between the two groups was determined by analysis of covariance.

### MTHFR C677T polymorphism

Allele and genotype frequencies in both LG and non-LG are described in Table 2. The genotypes within each group were distributed in accordance with the Hardy-Weinberg equilibrium. The overall prevalence of genotypes and alleles were significantly different between the two groups (*P* = 0.002 and 0.001, respectively), with T allele and TT genotype presenting more highly in LG than in non-LG. These differences persist in females but not in males after sex stratification. In LG, almost equal genotypic and allelic frequency of MTHFR C677T was observed between sexes, while in non-LG, men presented higher T allele and TT genotype than women.

**Table 2:** Distribution of MTHFR C677T genotype and allele in LG and non-LG

		**Genotype n (%)**			**Allele n (%)**	
**Group**	**N**	**CC**	**CT**	**TT**	**C**	**T**
All	1009	700 (69.38)	266 (26.36)	43 (4.26)	1666 (82.56)	352 (17.44)
LG	516	332 (64.34)	159 (30.81)	26 (4.84)	823 (79.75)	209 (20.25)
non-LG	493	368 (74.65)	107 (21.70)	18 (3.65)	843 (85.50)	143 (14.50)
χ^2^	-	12.639			11.573	
*P*	-	0.002			0.001	
LG						
Male	127	83 (65.35)	35 (27.56)	9 (7.09)	201 (79.13)	53 (20.87)
Female	389	249 (64.01)	124 (31.88)	16 (4.11)	622 (79.95)	156 (20.05)
χ^2^	-	2.353			0.079	
*P*	-	0.308			0.779	
non-LG						
Male	142	95 (66.90)	38 (26.76)	9 (6.32)	228 (80.28)	56 (19.72)
Female	351	273 (77.78)	69 (19.66)	9 (2.56)	615 (87.61)	87 (12.39)
χ^2^	-	7.896			8.750	
*P*	-	0.019			0.003	
Male						
LG	127	83 (65.35)	35 (27.56)	9 (7.09)	201 (79.13)	53 (20.87)
non-LG	142	95 (66.90)	38 (26.76)	9 (6.34)	228 (80.28)	56 (19.72)
χ^2^	-	0.096			0.109	
*P*	-	0.953			0.741	
Female						
LG	389	249 (64.01)	124 (31.88)	16 (4.11)	622 (79.95)	156 (20.05)
non-LG	351	273 (77.78)	69 (19.66)	9 (2.56)	615 (87.61)	87 (12.39)
χ^2^	-	16.830			15.771	
*P*	-	2.22E-4			7.15E-5	

### Genotypes and serum lipid levels

Lipid characteristics of participants by MTHFR genotype are presented in Table 3. In LG, non-LG and the pooled population, the difference in the four lipid measures among MTHFR C677T genotypes did not attain statistical significance under both co-dominant and T-allele dominant model (CT/TT). However, when sex was taken into account, T-allele carriers (CT/TT) of females but not males were found to have significantly greater level of TC and LDL-C than were non-T carriers (CC) in the entire population and in LG, indicating a possible detrimental nature of the C > T transition at 677 MTHFR in lipid modulation. Considering that hyperlipidemia may be more persuasive than lipid level in interpreting the association between the lipid metabolism and thrombophilic phenotypes, we further analyzed the influence of MTHFR C677T on lipids according to lipid status. Again, no significantly different lipid level was observed among the three genotypes in the hyperlipidemic and normolipidemic subgroup of LG and the hyperlipidemic subgroup of non-LG, with an exception of a moderate lower TC and lower LDL-C were noted in subjects harboring CT or TT genotype as compared with CC genotype in the normolipidemic subgroup of non-LG (p = 0.001 and 0.005, respectively, Table 4).

**Table 3:** Impact of MTHFR C677T on serum lipid levels (mmol/L)

**Group**	**Genotype**	**n**	**TC**	**TG**	**HDL-C**	**LDL-C**
**All**						
	CC	700	5.04 ± 0.97	0.94 (0.52)	1.66 ± 0.39	2.86 ± 0.90
	CT	266	5.15 ± 1.01	0.98 (0.53)	1.63 ± 0.39	3.01 ± 0.84
	TT	43	4.86 ± 1.31	0.88 (0.41)	1.61 ± 0.46	2.70 ± 0.98
	F	—	1.099	0.617	0.190	1.537
	*p*	—	0.334	0.540	0.827	0.215
	CC	700	5.04 ± 0.97	0.94 (0.52)	1.66 ± 0.39	2.86 ± 0.90
	CT/TT	309	5.11 ± 1.06	0.96 (0.50)	1.64 ± 0.41	2.97 ± 0.87
	F	—	0.163	1.335	0.022	0.001
	*p*	—	0.686	0.248	0.883	0.974
**Male**						
	CC	178	5.00 ± 1.04	0.95 (0.70)	1.61 ± 0.41	2.84 ± 0.96
	CT	73	4.78 ± 0.97	0.87 (0.48)	1.59 ± 0.42	2.72 ± 0.83
	TT	18	4.70 ± 0.98	0.83 (0.45)	1.68 ± 0.57	2.58 ± 0.73
	F	—	1.560	2.001	0.477	1.106
	*p*	—	0.212	0.137	0.621	0.332
	CC	178	5.00 ± 1.04	0.95 (0.70)	1.61 ± 0.41	2.84 ± 0.96
	CT/TT	91	4.77 ± 0.97	0.86 (0.46)	1.61 ± 0.45	2.69 ± 0.81
	F	—	3.042	3.837	0.025	1.830
	*p*	—	0.082	0.051	0.876	0.177
**Female**						
	CC	522	5.05 ± 0.95	0.93 (0.49)	1.68 ± 0.38	2.87 ± 0.88
	CT	193	5.29 ± 0.99	1.02 (0.62)	1.65 ± 0.38	3.12 ± 0.82
	TT	25	4.97 ± 1.52	1.01 (0.43)	1.61 ± 0.46	2.79 ± 1.13
	F	—	3.762	0.452	0.181	4.227
	*p*	—	0.024	0.637	0.835	0.015
	CC	522	5.05 ± 0.95	0.93 (0.49)	1.68 ± 0.38	2.87 ± 0.88
	CT/TT	218	5.26 ± 1.06	1.02 (0.53)	1.65 ± 0.39	3.08 ± 0.86
	F	—	4.183	0.766	0.232	4.504
	*p*	—	0.041	0.382	0.630	0.034
**LG**						
	CC	332	5.12 ± 1.00	0.97 (0.49)	1.59 ± 0.37	3.04 ± 0.87
	CT	159	5.22 ± 1.06	0.99 (0.50)	1.61 ± 0.38	3.09 ± 0.86
	TT	25	5.13 ± 1.31	1.07 (0.44)	1.56 ± 0.51	3.01 ± 0.96
	F	—	0.148	0.017	0.073	0.305
	*p*	—	0.863	0.983	0.930	0.737
	CC	332	5.12 ± 1.00	0.97 (0.49)	1.59 ± 0.37	3.04 ± 0.87
	CT/TT	184	5.20 ± 1.09	1.00 (0.48)	1.61 ± 0.40	3.08 ± 0.88
	F	—	0.276	0.003	0.000	0.558
	*p*	—	0.600	0.959	0.993	0.456
**Male**						
	CC	83	5.00 ± 1.11	0.94 (0.39)	1.57 ± 0.41	2.96 ± 0.95
	CT	35	4.67 ± 1.11	0.87 (0.29)	1.54 ± 0.39	2.67 ± 0.97
	TT	9	4.71 ± 1.09	0.84 (0.53)	1.55 ± 0.64	2.76 ± 0.77
	F	—	1.377	0.983	0.081	1.255
	*p*	—	0.256	0.377	0.922	0.289
	CC	83	5.00 ± 1.11	0.94 (0.39)	1.57 ± 0.41	2.96 ± 0.95
	CT/TT	44	4.67 ± 1.09	0.87(0.34)	1.54 ± 0.44	2.69 ± 0.92
	F	—	2.749	1.398	0.158	2.450
	*p*	—	0.100	0.239	0.691	0.120
**Female**						
	CC	249	5.15 ± 0.96	0.98 (0.52)	1.60 ± 0.35	3.06 ± 0.84
	CT	124	5.37 ± 1.00	1.04 (0.62)	1.63 ± 0.38	3.21 ± 0.80
	TT	16	5.37 ± 1.40	1.09 (0.47)	1.56 ± 0.44	3.15 ± 1.05
	F	—	2.179	2.148	0.605	1.188
	*p*	—	0.115	0.113	0.546	0.306
	CC	249	5.15 ± 0.96	0.98 (0.52)	1.60 ± 0.35	3.06 ± 0.84
	CT/TT	140	5.37 ± 1.04	1.07 (0.61)	1.63 ± 0.38	3.20 ± 0.83
	F	—	4.366	3.091	0.580	2.307
	*p*	—	0.037	0.080	0.447	0.130
**Non-LG**						
	CC	368	4.97 ± 0.95	0.91 (0.60)	1.73 ± 0.40	2.70 ± 0.90
	CT	107	5.06 ± 0.92	0.95 (0.59)	1.66 ± 0.41	2.90 ± 0.79
	TT	18	4.47 ± 1.25	0.85 (0.24)	1.75 ± 0.49	2.28 ± 0.86
	F	—	4.138	2.135	0.728	4.601
	*p*	—	0.017	0.119	0.483	0.010
	CC	368	4.97 ± 0.95	0.91 (0.60)	1.73 ± 0.40	2.70 ± 0.90
	CT/TT	125	4.98 ± 1.00	0.89 (0.47)	1.68 ± 0.42	2.81 ± 0.83
	F	—	0.137	1.833	0.032	0.128
	*p*	—	0.712	0.176	0.858	0.721
**Male**						
	CC	95	4.99 ± 0.99	0.97 (0.91)	1.63 ± 0.41	2.75 ± 0.96
	CT	38	4.89 ± 0.83	0.89 (0.70)	1.64 ± 0.45	2.76 ± 0.68
	TT	9	4.69 ± 0.92	0.81 (0.49)	1.81 ± 0.49	2.40 ± 0.68
	F	—	0.677	1.372	1.096	0.963
	*p*	—	0.510	0.257	0.337	0.384
	CC	95	4.99 ± 0.99	0.97 (0.91)	1.63 ± 0.41	2.75 ± 0.96
	CT/TT	47	4.86 ± 0.84	0.85 (0.67)	1.67 ± 0.46	2.69 ± 0.69
	F	—	0.670	2.637	0.537	0.212
	*p*	—	0.415	0.107	0.465	0.646
**Female**						
	CC	273	4.96 ± 0.94	0.90 (0.52)	1.76 ± 0.40	2.69 ± 0.88
	CT	69	5.15 ± 0.96	1.00 (0.49)	1.68 ± 0.38	2.97 ± 0.84
	TT	9	4.26 ± 1.54	0.85 (0.24)	1.70 ± 0.52	2.16 ± 1.05
	F	—	5.413	1.114	0.820	5.323
	*p*	—	0.005	0.330	0.441	0.005
	CC	273	4.96 ± 0.94	0.90 (0.52)	1.76 ± 0.40	2.69 ± 0.88
	CT/TT	78	5.05 ± 1.07	0.95 (0.40)	1.68 ± 0.40	2.88 ± 0.90
	F	—	0.287	0.063	1.727	1.594
	*p*	—	0.592	0.803	0.190	0.208

**Table 4:** Impact of MTHFR C677T on serum lipid levels according to lipid status (mmol/L)

**Group**	**Genotype**	**n**	**TC**	**TG**	**HDL-C**	**LDL-C**
**LG**						
Dyslipidemia						
	CC	167	5.84 ± 0.76	1.14 (0.69)	1.68 ± 0.39	3.57 ± 0.74
	CT	84	5.96 ± 0.75	1.16 (0.71)	1.70 ± 0.41	3.66 ± 0.70
	TT	10	6.30 ± 1.14	1.13 (1.10)	1.72 ± 0.66	3.80 ± 0.89
	F	—	1.265	0.120	0.501	0.431
	*p*	—	0.284	0.887	0.607	0.605
	CC	167	5.84 ± 0.76	1.14 (0.69)	1.68 ± 0.39	3.57 ± 0.74
	CT/TT	89	5.99 ± 0.81	1.16 (0.72)	1.71 ± 0.44	3.67 ± 0.72
	F	—	1.001	0.041	0.232	0.625
	*p*	—	0.318	0.839	0.631	0.430
Normolipidemia						
	CC	165	4.38 ± 0.58	0.85 (0.33)	1.51 ± 0.32	2.49 ± 0.62
	CT	75	4.38 ± 0.65	0.85 (0.28)	1.51 ± 0.32	2.45 ± 0.52
	TT	15	4.35 ± 0.69	0.94 (0.47)	1.45 ± 0.36	2.48 ± 0.57
	F	—	0.021	0.228	0.240	0.222
	*p*	—	0.979	0.796	0.786	0.801
	CC	165	4.38 ± 0.58	0.85 (0.33)	1.51 ± 0.32	2.49 ± 0.62
	CT/TT	90	4.38 ± 0.65	0.85 (0.34)	1.50 ± 0.33	2.46 ± 0.53
	F	—	0.029	0.350	0.109	0.149
	*p*	—	0.865	0.555	0.742	0.700
**Non-LG**						
Dyslipidemia						
	CC	168	5.65 ± 0.92	1.16 (0.98)	1.71 ± 0.45	3.19 ± 1.00
	CT	53	5.77 ± 0.59	1.11 (1.06)	1.68 ± 0.43	3.43 ± 0.61
	TT	6	5.85 ± 0.39	0.85 (0.55)	2.19 ± 0.49	3.12 ± 0.46
	F	—	0.307	1.270	1.880	0.501
	*p*	—	0.736	0.283	0.155	0.606
	CC	146	5.71 ± 0.66	1.16 (0.98)	1.71 ± 0.41	3.43 ± 0.64
	CT/TT	58	5.76 ± 0.58	1.08 (1.13)	1.70 ± 0.42	3.45 ± 0.58
	F	—	−0.530	−0.443	0.121	−0.148
	*p*	—	0.597	0.658	0.903	0.883
Normolipidemia						
	CC	200	4.40 ± 0.50	0.80 (0.34)	1.74 ± 0.36	2.29 ± 0.52
	CT	54	4.36 ± 0.58	0.69 (0.32)	1.65 ± 0.39	2.37 ± 0.56
	TT	12	3.79 ± 0.90	0.79 (0.24)	1.53 ± 0.33	1.86 ± 0.70
	F	—	7.662	0.987	1.272	5.337
	*p*	—	0.001	0.374	0.282	0.005
	CC	200	4.40 ± 0.50	0.80 (0.34)	1.74 ± 0.36	2.29 ± 0.52
	CT/TT	66	4.26 ± 0.68	0.75 (0.33)	1.63 ± 0.38	2.27 ± 0.61
	F	—	1.478	1.202	1.339	0.156
	*p*	—	0.225	0.274	0.248	0.694

### Correlation between serum lipid parameters and genotypes

Multiple linear regression analyses showed that TC, TG and LDL-C were positively while HDL-C was negatively correlated in the main with age, diastolic blood pressure and BMI but not with MTHFR C677T genotypes in the overall population, i.e. the older of age and the higher of diastolic blood pressure and BMI, the higher of unfavorable lipids (Table 5). After sex stratification, lipids, TC and TG in particular, correlated with not only the above mentioned factors, but also with MTHFR C677T genotypes in both sexes. When LG and non-LG were analyzed separately, the correlation between lipids and MTHFR C677T genotype exist mainly in the females of LG. These findings are basically in line with that in Table 1 and Table 3.

**Table 5:** Correlation between serum lipid parameters and the MTHFR C677T polymorphism

**Group/Lipid**	**Relative factor**	**Unstandardized coefficients**		**Standardized coefficients**	***t***	***P***
		**B**	**Std. error**	**Beta**		
**All**						
TC	DBP	0.010	0.003	0.123	3.802	1.52E-4
	BMI	0.035	0.009	0.121	3.794	1.57E-4
	Sex	0.207	0.070	0.091	2.960	0.003
	Age	0.005	0.002	0.067	2.073	0.038
TG	BMI	0.016	0.002	0.265	8.754	8.59E-18
	SBP	0.001	0.000	0.094	3.099	0.002
HDL-C	Age	−0.005	0.001	−0.173	−5.532	4.04E-8
	BMI	−0.018	0.004	−0.156	−4.992	7.03E-7
	Sex	0.068	0.028	0.076	2.437	0.014
LDL-C	Age	0.011	0.002	0.169	5.247	1.88E-7
	DBP	0.008	0.002	0.115	3.599	3.35E-4
	BMI	0.027	0.008	0.102	3.228	0.001
	Sex	0.137	0.062	0.068	2.230	0.026
**Male**						
TG	BMI	0.010	0.004	0.157	2.593	0.010
	Age	−0.002	0.001	−0.142	−2.343	0.020
	Genotype	−0.042	0.020	−0.127	−2.136	0.034
HDL-C	BMI	−0.029	0.008	−0.225	−3.700	2.62E-4
	Age	−0.005	0.002	−0.153	−2.522	0.012
**Female**						
TC	DBP	0.009	0.003	0.117	3.052	0.002
	BMI	0.046	0.011	0.160	4.311	1.85E-5
	Age	0.009	0.003	0.118	3.078	0.002
TG	BMI	0.019	0.001	0.327	9.317	1.35E-19
	Age	0.002	0.001	0.169	4.822	1.73E-6
HDL-C	Age	−0.005	0.001	−0.183	−5.013	6.71E-7
	BMI	−0.015	0.004	−0.132	−3.601	3.38E-4
LDL-C	Age	0.013	0.002	0.199	5.176	2.94E-7
	BMI	0.030	0.009	0.117	3.184	0.002
	DBP	0.007	0.003	0.105	2.759	0.006
	Genotype	0.139	0.069	0.072	2.006	0.045
**LG**						
TC	DBP	0.012	0.003	0.160	3.699	3.40E-4
	Sex	0.323	0.103	0.135	3.130	0.002
TG	BMI	0.007	0.002	0.160	3.708	2.32E-4
	Sex	0.046	0.016	0.120	2.779	0.006
HDL-C	BMI	−0.015	0.005	−0.145	−3.312	0.001
	DBP	0.001	0.001	0.103	2.359	0.019
LDL-C	DBP	0.008	0.003	0.124	2.857	0.004
	Sex	0.232	0.088	0.115	2.639	0.009
**Male**						
TC	DBP	0.015	0.007	0.181	2.056	0.042
HDL-C	BMI	−0.026	0.011	−0.217	−2.480	0.014
LDL-C	DBP	0.014	0.006	0.200	2.277	0.024
**Female**						
TC	DBP	0.011	0.004	0.154	3.072	0.002
	Genotype	0.215	0.103	0.104	2.087	0.038
TG	BMI	0.029	0.007	0.197	3.974	8.45E-5
	Genotype	0.117	0.047	0.124	2.511	0.012
HDL-C	Age	0.014	0.006	0.111	2.204	0.028
**Non-LG**						
TC	BMI	0.081	0.013	0.263	6.047	2.92E-9
TG	BMI	0.097	0.013	0.324	7.598	1.54E-13
HDL-C	BMI	−0.019	0.006	−0.149	−3.314	0.001
	SBP	−0.002	0.001	−0.118	−2.636	0.009
	Sex	0.084	0.040	0.094	2.133	0.033
LDL-C	BMI	0.067	0.013	0.237	5.304	1.72E-7
	Age	0.022	0.008	0.120	2.691	0.007
**Male**						
TC	BMI	0.061	0.027	0.189	2.307	0.023
	Age	−0.046	0.020	−0.186	−2.265	0.025
TG	Age	−0.064	0.021	−0.247	−3.066	0.003
	BMI	0.064	0.027	0.188	2.336	0.021
HDL-C	BMI	−0.033	0.012	−0.222	−2.698	0.008
LDL-C	BMI	0.064	0.025	0.212	2.569	0.011
**Female**						
TC	BMI	0.087	0.016	0.285	5.553	5.57E-8
TG	BMI	0.103	0.014	0.364	7.294	2.03E-12
HDL-C	SBP	−0.003	0.001	−0.166	−3.126	0.002
	BMI	−0.015	0.007	−0.121	−2.281	0.023
LDL-C	BMI	0.060	0.015	0.216	3.983	8.28E-5
	Age	0.029	0.009	0.162	3.093	0.002
	DBP	0.012	0.004	0.139	2.632	0.009

Note: DBP, Diastolic blood pressure; SBP, Systolic blood pressure; BMI, Body mass index.

## Discussion

In the present study, the prevalence of MTHFR 677 T was 17.44% and 14.50% in the combined samples and control population respectively, with a lower frequency of TT homozygote (≈4%). These allelic and genotypic frequencies are close to that of Bai Ku Yao (22.6%) whereas much lower than that of Guangxi Han Chinese (39.1%), two neighboring populations we reported recently [18].

It has been noted that the distribution of the T allele vary substantially across general populations worldwide, with a north-to-south increase in European continent but a reverse gradient in China mainland [19-21]. Zhuang and Bai Ku Yao are typical aboriginal ethnic groups in Southern China, our results thus defer somewhat to this pattern. The underlying mechanism for this great geographical diversity remains unclear, adaptation to external conditions such as climate or nutritional status could be one explanation [22]. Further investigation of gene-gene and gene-environment interaction might help to determine the evolutionary pressures favouring a high prevalence of this variant in certain areas and ethnic groups.

Intriguingly, MTHFR 677 T and its relevant genotypes (CT, TT) were significantly more prevalent in our nonagenarian and centenarian populations, especially in females, as compared with the elder control group (60–75 years). These observations implicate a potential association between MTHFR C677T polymorphism and longevity in Bama area. A question of whether this variant is favorable or deleterious for Bama long-lived individuals therefore emerges. According to most but not all reported data, MTHFR 677 T genotypes have been linked to unfavorable lipid profiles, including greater concentrations of TC, TG, and LDL-C [18,23-25] and lower level of HDL-C [26,27], all known risk factors for cardiovascular and metabolic diseases. Herein, the female but not male T-allele carriers in the pooled population and in LG presented greater level of TC, TG and LDL-C than did T noncarriers (Table 3 and Table 5), in agreement with prevailing data as aforementioned, indicating that MTHFR 677 T genotypes may play detrimental rather than beneficial role in lipid modulation and survivorship. However, this influence seems to be limited because the impact of MTHFR 677 T on lipid metabolism remained only in the normolipidemic subgroup of non-LG after further analysis according to lipid status categorization (Table 4). Together, these data suggest that although MTHFR C677T affects the TC and LDL-C metabolism of LG and the overall population studied to varying degree, particularly of the females in LG, these impacts may not be sufficient to cause extremely lipid abnormality.

With regard to the enrichment of deleterious genotypes in long-lived females, it might be partially interpreted by the Buffering Mechanisms in Aging hypothesis proposed recently by Bergman and colleagues who reasoned that in a subpopulation endowed with a favorable longevity genotype(s), the prevalence of a deleterious genotype is expected not to vary or even increase with age because the longevity genotype may buffer out or modulate the harmful effect of deleterious ones, while in a subpopulation lacking longevity genotypes, the prevalence of a deleterious genotype will decrease with age since subjects with this genotype are weeded out due to mortality [28,29]. Therefore, screening for potential longevity genotypes such as CETP (VV) (rs5882), APOC3 (CC) (rs2542052), AdipoQ (del/del APM1 + 2019) (rs56354395), and FOXO3a (GG), which have been demonstrated in other populations [30-33], will be one of our next efforts in the near future.

Due to the established link among MTHFR C677T polymorphism, Hcy, cardiovascular risk and aging, several investigations have been conducted in some elder cohorts to look at the possible contribution of MTHFR to longevity. However, findings are still inconsistent thus far. For instance, lower frequency of MTHFR C677T +/+genotype, with raised Hcy level, was observed in French cohort with longevity trait (> 90 yrs, n = 564) than in controls (< 70 yrs, n = 374), albeit no statistical significance was reached [34]. Similar observations were made in Swiss (106 elderly, 68–95 yrs vs 118 younger, 21–64 yrs) [35], Japanese (148 oldest, > 80 yrs including 22 nonagenarians vs 311 younger, < 55 yrs) [36] and Jerusalem Ashkenazi (224 elderly, > 75 yrs vs 441 controls, < 22 yrs) [37] populations, while almost equal prevalence of MTHFR C677T genotypes were seen between elderly and younger group in Swede (222 elderly, 80–108 yrs vs 220 newborn) [38], British (282 elderly, > 84 yrs vs 200 younger, < 17 yrs) [39], and Jordanian (130 elderly, > 85 yrs, mean age 90.01 yrs vs 135 younger, 20–50 yrs, mean age 33.34 yrs) [40] population. Conversely, we detected a higher prevalence of MTHFR 677 T in our oldest olds. Although it is known that MTHFR TT might be a risk biomarker against longevity from other perspectives, we cannot exclude the possibility that it may also be in favor of good health or long life span as implicated by Le Marchand et al. that there might be an inverse association between MTHFR 677TT genotype and the development of colorectal cancer [41]. To the best of our knowledge, the nonagenarian population here is the only long-lived cohort that enriches this variant, whose significance deserves further clarification. These discrepancies may arise from factors related to differences in ethnic background of the population studied, recruitment strategy for long-lived and control group, assessment methods or sample sizes.

Despite strengths such as large sample size, highly population genetic homogeneity and well-characterized cases and controls, all the difference of lipid profiles between long-lived individuals and controls cannot completely attribute to MTHFR C677T. The current study should be viewed in the light of some limitations: (1) no determination of folate status, serum MTHFR activity and Hcy levels on baseline data collection due to insufficient funding, which would be more significantly conclusive and would help to interpret the selection and the outcome of MTHFR 677 T [22,42]; (2) lack of the evaluation of other modifying genetic variants, risk factors such as smoking and alcohol consumption and lifestyle which may interact with MTHFR C677T and change its association with longevity; (3) we could not completely exclude asymptomatic disorders such as atherosclerosis which may create a potentially significant bias due to poor field study condition; (4) it is very much speculative to imagine the controls would have a short life span because of a specific family history, this may also be a significant bias factor. (5) longitudinal follow up is warranted in further interpreting the potential effect of MTHFR C677T polymorphism on lipid metabolism.

## Conclusions

Overall, our results show that MTHFR 677 T allele and TT genotype are accumulative in Bama long-living individuals in a gender-specific manner and are associated with unfavorable lipid profile although this impact on lipid modulation seems limited. Potential longevity gene(s) which may interact with or buffer out the deleterious effect of this variant needs to be determined.

## Competing interests

The authors declare that they have no competing interests.

## Authors’ contributions

NYC participated in the design, undertook genotyping, and drafted the manuscript. CWL, LLD, LPX, LG, YYW and ZW helped with genotyping. HYW, CYL, LL, JHP, XQL, RXY and CPN took part in the epidemiological survey and sampling. SLP conceived the study, participated in the design, carried out the epidemiological survey, collected the samples, and helped to draft the manuscript. All authors read and approved the final manuscript.
